# Nursing in Workhouse Infirmaries

**Published:** 1887-03-19

**Authors:** Samuel Benton


### Nursing in Workhouse Infirmaries.

By Samuel Benton, M.R.C.S.

Since the conference on Trained Nursing in Workhouse Infirmaries, held at the Marylebone Infirmary, 8th December 1881, many advances in the right direction have been made, due in part to the untiring efforts of the Workhouse Infirmary Nursing Association, which has just issued its seventh annual report. In my Paper read on that occasion stress was laid upon the advantages that would accrue to the sick poor, if the clause in Gathorne Hardy's Act, which hinders workhouse infirmaries from being utilised for the clinical study of disease, were amended. This subject has been most ably reopened by Colonel Montefiore, and will now no doubt bear fruit.

**The "Lancet" Commission.**

In the spring of 1880 *The Lancet* appointed a commission to inquire into the working of the new sick-infirmaries attached to the Metropolitan workhouses. The Commissioners pointed out that the medical nursing staff fell very short of the requirements for efficiently dealing with cases of acute sickness arising among the poor. The Commissioners expressed an opinion that the medical staff should be strengthened by the appointment of clinical assistants, and thus, by relieving the medical superintendent of much unnecessary work, permit him to give more attention to serious and urgent cases. They also recommended that the nursing staff should be largely increased.

**Ratepayers to be Primarily Considered.**

In the administration of the Poor-law the ratepayers ought to be primarily considered. Now I ask, is there any economy in huddling a lot of sick people together (very many of them curable cases) in an infirmary which is inefficiently officered, by only two medical men, and with nurses untrained and overworked? I will only quote one out of hundreds of curable cases which came under my notice some years ago. A working man, who was admitted to the Central London Sick Asylum at Highgate, suffering from acute pneumonia, at the end of three weeks was discharged well—a fact due largely to his being nursed by trained, trustworthy women. During his stay in the infirmary his wife and five children were in the workhouse, where they would have remained, a burden on the ratepayers for years, if the man's life had not been saved by good and efficient nursing. When the breadwinner is prostrated by disease or bodily injury it is advantageous to the ratepayer that he be rapidly restored to health and strength, so that he can return to his labour, and cease to be a burden upon the rates. That no infirmary can be efficient without a proper staff of trained nurses is becoming daily more and more apparent. Even from the financial point of view the necessity is strongly urgent, and the Guardians must ere long become conscious of this, and willing to authorise the primary outlay to procure this favourable result. As our voluntary hospitals are languishing for want of funds, and are obliged in some cases to make a charge to the patients towards their maintenance, this must drive a greater number of acute and chronic cases into our State infirmaries.

**Deficient Number of Medical Officers.**

Many of the new infirmaries are built to accommodate six hundred or more sick people, for if they are only infirm they ought to be in the workhouse. Two medical men are expected to do all this work, and in some infirmaries the assistant is further required to dispense the medicines. Five minutes does not seem an unreasonable time to give each patient, but if the doctors were to give each patient five minutes' attention, they would be twenty-five hours going round the wards.

**Infirmary Nursing will bear favourable Comparison with Hospital Nursing.**

It has been stated by some that infirmary nursing is not popular, therefore it is difficult to secure the better class of women to take up this work. This, I must say, from personal experience, is not always the case. I have known women of the highest order of nurses who preferred infirmary to hospital nursing. It must be borne in mind that the minor dressing and even note-taking which is performed in hospitals by the students, in the infirmaries fall to the duty of the charge nurses. If nurses are discontented with their work, it may be accounted for in some instances by the medical officers being overworked, and unable to interest the nurses in their work. When clinical clerks are freely admitted to our infirmaries, as they ought to have been long since, and the cases properly sorted and arranged, this will help considerably to improve the nursing. It will also be a great boon to the sick poor; there will then be less chance of acute serious cases being overlooked, and chronic cases passed by month after month deemed irremediable. The workhouse infirmaries as at present administered do not render such services to medical science and education as they might.

**Separation of Workhouses from Infirmaries.**

It is important that the infirmaries be effectually severed from the workhouses and the workhouse officers. In the first place, the sick should be entirely separated from the able-bodied. Pauper nurses are gradually being done away with, but they are still to be found in some Unions. There are infirmaries in which a paid nurse will be found in charge of 150 beds with pauper help. When we consider how important a factor sickness is amongst the working classes in the production of pauperism, there is no economy in having the sick inefficiently nursed. For the sick poor we need infirmaries where the maladies which pauperise the humbler classes, and throw their families too often permanently on the rates, may be promptly and efficiently treated.

**Each Infirmary should Train Nurses for its own use.**

At a rough estimate there might be one probationary nurse to every eighty beds. We hope this plan will be adopted in the course of time at all the large infirmaries; but at present sufficient building accommodation is not provided for the staff. At some of our new infirmaries, rooms for the nurses have had to be built, as an after-thought, in some dismal corner. It is not unnatural that these mistakes should have arisen. The class of men in the past who have given their time gratuitously to act as Guardians are not all the kind of men one would expect to know anything about nursing or hospital management; but these men are ready to admit the reforms which public opinion has imperatively called for. The Workhouse Nursing Association has endeavoured to arouse public opinion to the necessity of reforms, especially in the nursing arrangements. Of late years many ladies have come forward and been elected as Guardians. When we consider the number of women and children in our workhouses, the presence of ladies on the Board has proved most valuable.

Probationers.

In the large infirmaries the vacancies in the nursing staff can be filled up by the probationary nurses. There is a great demand for night nurses, the salary being in some cases lower than that offered for day work, and some infirmaries have great difficulty in getting first-rate nurses to take night-work. This difficulty, I think, might be partly met by insisting on probationary nurses, just out of their time, taking, say, six months' night-duty before being given charge of a ward. It is right that a matron and night-superintendent of nurses should have had hospital experience, but, provided an infirmary is large enough, the ordinary nurses can be quite well trained in all branches of nursing in a workhouse infirmary. It is remarkable, as a proof of how little the future of infirmaries was contemplated, when rules were issued by the Local Government Board, that no allusion is made to the supervision of *nursing* by the matron, the duties being merely supposed to be those of a housekeeper. This oversight leaves the appointments open to inferior officials. There is a large demand for trained nurses in our country workhouse infirmaries, and they are too small to train their own nurses. In sending trained nurses into the country, one difficulty is their having to work under untrained matrons; another difficulty is that the woman appointed should be a certified midwife as well as a trained nurse, there being very seldom a resident medical officer.

Midwifery Training.

I am indebted to Miss Wilson, the indefatigable Honorary Secretary to the Workhouse Infirmary Nursing Association, for many valuable hints for this Paper, and she has drawn especial attention to the question of midwifery training for country workhouse nurses. The Association has lately commenced to train midwives. Quoting from the seventh and last report, I notice: "By permission of the Kensington Board of Guardians, we have entered into an arrangement by which we are entitled to train a regular number of probationers yearly in the maternity wards of their infirmary. This successful beginning at Kensington causes us to hope that the same plan may be extended to other infirmaries. The demand from country workhouses for trained nurses with the additional qualification continues larger than we can supply, and we are anxious to increase the field for training. Eight probationers are in training, viz., Brownlow Hill Infirmary, Liverpool, 3; Crumpsall Infirmary, Manchester, 1; Salisbury Infirmary, 1; Chichester Infirmary, 2; Kensington Infirmary (maternity ward), 1." To obviate the first difficulty, in the country it is desirable a higher class of matron and master should be appointed. The second difficulty in these appointments, the need for midwifery training, can only be obviated by the larger infirmaries throwing open their lying-in wards for purposes of training, *at a moderate fee*. The present fees at lying-in hospitals vary from £20 to £26; at Brownlow Hill Infirmary, Liverpool, £10; and Crumpsall Infirmary, Manchester, £15, for a course of three months. The Kensington Infirmary is the only infirmary in London which at present trains in *practice* and *theory*. Lambeth Infirmary allows several outside pupils to come for *the cases only*. No lectures or classes are, however, given there. The experience is excellent in these large infirmaries. In 1886, 70 cases in three months was the average at Brownlow Hill; number at Marylebone, 212; at Kensington, 132; St Pancras, 184.

The Matron a Trained Lady Nurse.

No woman ought to be appointed to the position of matron to a workhouse infirmary who is not a lady and a trained nurse. The mistake of promoting a woman to the position of matron to a new infirmary, who has no previous experience than that of a workhouse nurse or matron, has been tried, and the failure is most appalling. It is impracticable to send a young and carefully trained nurse, just out of her probation, to fill the vacancy advertised by some infirmary where the nursing is indifferent and the matron untrained. To satisfy the incredulous this experiment has been again tried, with the hope that by the zeal with which she discharges her duties, the way may be gradually paved for better things. Of course it failed; the trained nurse was unable to battle against the difficulties with which she had to contend. From the first the matron looked on her with a jealous eye. It is hopeless to send a trained nurse to an infirmary under an untrained matron. That there is still need for further efforts in this direction is shown from the statement taken from a report of London infirmaries, drawn up during the year by Colonel Montefiore for the Charity Organisation Society. In the twenty-seven Metropolitan infirmaries—fourteen of which are well built on the pavilion, *i.e.*, separate-block system—it is stated that only thirteen of the matrons are trained. Further attention has been drawn to the needs of Poor-law reform by Miss Louisa Twining, in an article in the November number of the *Nineteenth Century*, in which many examples are given of the drawbacks involved in the employment of untrained or pauper nurses. Quoting from the last report of the Workhouse Infirmary Nursing Association:—"People are perhaps scarcely aware that many of the London workhouses still contain a large number of sick for whom the nursing and the general arrangements are often very inadequate. Having made the attempt in five instances to place trained nurses under these conditions, we have been obliged to relinquish the endeavour, owing principally to the following difficulties: (*a*) the authority of an untrained matron, unacquainted with the needs of the sick, and the consequent friction between *untrained* matron and *trained* nurse, produced by the requirements of a nurse for her patients; (*b*) the very inadequate number of paid nurses employed, and as a result the employment of pauper assistance; (*c*) in some cases the unsatisfactory arrangements for the nurses' rooms and meals."

Recreation and Diet.

We consider it a pernicious custom to allow young nurses to take their off time in the evening after dark. A matron who is a good manager can arrange for her nurses to take their half-day off during the afternoon, and occasionally—say, once a month—give them a whole day's holiday to enable them to visit their friends at a distance. Not sufficient attention is paid in many infirmaries to the diet of the nurses. The cooking ought to be good, and the diet not only ample, but varied. Boiled mutton four days a week, without sufficient change of vegetables, is rather trying. In the spring and summer time a salad might be given. Puddings and sweets should be served daily; they are not so expensive as meat, and are usually even more appreciated by women than men. The nurses' sleeping-rooms ought to be placed in a quiet part of the building, and kept free from noise. Where entirely separate rooms are impossible, cubicles are desirable.

Uniform for Nurses.

It is important that the uniform supplied to nurses should be neat and inexpensive. Upon inquiry, it is found that those infirmaries which supply by contract, both outdoor and indoor uniforms, pay but a few shillings more per annum than others which only give indoor uniforms.

A moment's reflection will show the advantage it gives a matron in maintaining discipline if the nurses have outdoor uniforms. In the paper written for this Association last month by the Matron of St. Bartholomew's Hospital, it is inferred by Miss Manson that "women always live up to their outward appearance." Guardians are naturally very jealous in the exercise of their right in selecting and appointing their own officers. When we consider that in our new infirmaries the majority of women employed in the responsible position of matron are untrained, it behoves every ratepayer to bestir himself. This state of things has been brought about, in some instances by the Guardians fearing that they may be called upon to pension old servants, and in other instances by a desire to find employment for poor relations or friends. It is very necessary to be careful in the selection of old workhouse officers to new infirmaries; they are apt to spoil the whole tone of the institution and to retard modern reforms.

